# 电磁导航支气管镜在肺外周病变中诊断价值及安全性的*meta*分析

**DOI:** 10.3779/j.issn.1009-3419.2023.102.07

**Published:** 2023-02-20

**Authors:** Xin SUN, Yu SU, Shangyao LI, Yu TIAN, Liang ZHAO

**Affiliations:** ^1^100021 北京，国家癌症中心/国家肿瘤临床医学研究中心/中国医学科学院北京协和医学院肿瘤医院（孙鑫，赵亮）; ^1^National Cancer Center/National Clinical Research Center for Cancer/Cancer Hospital, Chinese Academy of Medical Sciences and Peking Union Medical College, Beijing 100021, China; ^2^100069 北京，首都医科大学公共卫生学院（苏宇，李尚尧，田雨）; ^2^School of Public Health, Capital Medical University, Beijing 100069, China

**Keywords:** 电磁导航支气管镜, 肺外周病变, 肺结节, 诊断价值, Meta分析, Electromagnetic navigation bronchoscopy, Peripheral pulmonary lesions, Pulmonary nodules, Diagnostic value, Meta-analysis

## Abstract

**背景与目的:**

肺癌的发病率与死亡率一直位居恶性肿瘤前列。随着肺癌检测技术的进步与检测率的提高，检测技术对肺外周病变（peripheral pulmonary lesions, PPLs）的诊断准确率一直存在争议。本研究旨在系统评价电磁导航支气管镜（electromagnetic navigation bronchoscopy, ENB）在诊断PPLs中的诊断价值及安全性。

**方法:**

系统检索万方数据知识服务平台、中国知网、Embase、PubMed、Cochrane Library、Web of Science关于ENB对PPLs诊断率的相关文献，应用Stata 16.0、RevMan 5.4与Meta-Disc 1.4软件进行meta分析。

**结果:**

共有54篇文献、合计55项研究被纳入数据分析。ENB在PPLs诊断中的合并灵敏度、特异度、阳性似然比、阴性似然比和诊断比值比分别为0.77（95%CI: 0.73-0.81）、0.97（95%CI: 0.93-0.99）、24.27（95%CI: 10.21-57.67）、0.23（95%CI: 0.19-0.28）和104.19（95%CI: 41.85-259.37）。受试者工作特征曲线下面积为0.90（95%CI: 0.87-0.92）。亚组分析与meta回归显示研究的异质性来源于研究类型、辅助定位技术、样本量、病变大小和麻醉类型。辅助定位技术和全身麻醉方式的使用提高了ENB在PPLs中的诊断效果。ENB操作相关的不良反应和并发症发生率较低。

**结论:**

ENB在PPLs诊断中具有良好的准确性与安全性。

## Abstract (en)

**Background and objective:**

The incidence and mortality of lung cancer have always been at the forefront of malignant tumors. With the development of lung cancer detection techniques, more peripheral pulmonary lesions (PPLs) have been detected. The diagnostic accuracy of procedures for PPLs keeps controversial. This study aims to systematically evaluate the diagnostic value and the safety of electromagnetic navigation bronchoscopy (ENB) in the diagnosis of PPLs.

**Methods:**

The relevant literatures in the diagnostic yield of PPLs by ENB were systematically retrieved from Wanfang Data Knowledge Service Platform, China National Knowledge Infrastructure, Embase, PubMed, Cochrane Library and Web of Science. The software of Stata 16.0, RevMan 5.4 and Meta-disc 1.4 were used to conduct the meta-analysis.

**Results:**

A total of 54 literatures with 55 studies were included in our meta-analysis. The pooled sensitivity, specificity, positive likelihood ratio, negative likelihood ratio and diagnostic odds ratio of ENB in the diagnosis of PPLs were 0.77 (95%CI: 0.73-0.81), 0.97 (95%CI: 0.93-0.99), 24.27 (95%CI: 10.21-57.67), 0.23 (95%CI: 0.19-0.28) and 104.19 (95%CI: 41.85-259.37), respectively. The area under curve (AUC) was 0.90 (95%CI: 0.87-0.92). Meta-regression and subgroup analyses indicated that the potential heterogeneity resulted from study type, additional localization techniques, sample size, lesion size and type of sedation. The use of additional localization techniques and general anesthesia have improved the diagnostic efficiency of ENB in PPLs. The incidence of adverse reactions and complications associated with ENB was very low.

**Conclusion:**

ENB provides well diagnostic accuracy and safety.

癌症已成为全球重大的公共卫生问题之一，给社会造成了严重的疾病负担。数据^[1,2]^表明，肺癌位居全球癌症发病第二位和死亡第一位，而在我国均为首位。目前肺癌缺乏病因学预防手段，筛查是早期发现肺癌的重要途径，而早发现和早诊断是提前知晓肺癌分期、降低肺癌死亡率的基础^[3]^。病理活检是肺癌诊断的金标准，但由于多数肺结节患者病变位置靠近外周、尺寸较小或超过常规支气管镜检查视野，因此既往对于肺外周结节的诊断主要依靠经胸壁穿刺肺活检[计算机断层扫描（computed tomography, CT）引导或超声引导]、手术活检或内镜取样，但三种方式各有利弊，其中手术活检创伤过大，穿刺活检并发症发生率较高，内镜取样的诊断准确率较低^[4]^。

近年来，电磁导航支气管镜（electromagnetic navigation bronchoscopy, ENB）逐渐在肺癌诊断中应用，其以电磁定位为基础，同时结合计算机虚拟支气管镜与高分辨率螺旋CT的特点，既可对传统支气管镜无法到达的肺外周病变（peripheral pulmonary lesions, PPLs）进行准确实时导航定位，又可对病变组织进行病理活检，还可直接进行消融等介入治疗^[5]^。ENB技术的出现为肺外周小结节提供了更加精确的定位、定性手段，但目前ENB诊断结果受到许多因素的影响，包括：（1）病变的特征，如病变大小、肺叶位置、有无支气管充气征、结节至胸膜距离^[6]^；（2）是否结合常用辅助技术，包括透视检查（fluoroscopy）、径向探头支气管内超声（radial endobronchial ultrasound, rEBUS）及快速现场评价（rapid on-site cytological evaluation, ROSE）^[6]^。目前国内外研究中ENB对于肺结节诊断效能的评价不一，因此本研究对全球范围内肺结节诊断相关文献进行meta分析，评估ENB对PPLs的诊断价值和安全性，以期为后续研究奠定基础。

## 1 资料与方法

### 1.1 资料来源

系统检索万方数据知识服务平台、中国知网、Embase、PubMed、Cochrane Library、Web of Science数据库，并且手工检索通过引文追溯获得的相关文献。语种限定为中文和英文。计算机检索时限均为建库至2022年12月。

### 1.2 文献检索式

英文：((electromagnetic navigation bronchoscopy) AND (diagnostic yield)) AND ((lung cancer) OR (pulmonary nodule) OR (peripheral lung lesions))，中文：[“电磁导航支气管镜”和“诊断率”和（“肺癌”或“肺结节”或“肺外周病变”）]。

### 1.3 文献筛选

在初步筛选文献时，制定统一的筛选流程与说明，利用EndNote X9软件对检索到的文献进行管理，排除重复文献。筛选文献时，阅读题目及摘要，在排除不相关文献后，进行全文阅读，以确定最终是否被纳入。使用诊断试验准确性研究的偏倚评估工具QUADAS-2量表对纳入文献进行全面质量评价。由两位研究者严格按照纳入和排除标准独立进行文献筛选、信息提取及质量评价，并交叉核对，如有意见不统一，则经共同讨论决定或交由第三人协助裁定。

#### 1.3.1 纳入标准

入选的PPLs患者均有影像学证据证实；ENB用于诊断PPLs；诊断经组织学或密切临床随访证实；有明确参考标准建立诊断准确性研究；语种限定为中文或英文。

#### 1.3.2 排除标准

与ENB或ENB的诊断率无关的文献；与肺结节或PPLs无关的文献；文章数据不完整或不可用；翻译版本或限定语种之外的版本；会议摘要或无全文的文献；综述类文章；个案报告；重复文献。最终共纳入文献54篇^[7⇓⇓⇓⇓⇓⇓⇓⇓⇓⇓⇓⇓⇓⇓⇓⇓⇓⇓⇓⇓⇓⇓⇓⇓⇓⇓⇓⇓⇓⇓⇓⇓⇓⇓⇓⇓⇓⇓⇓⇓⇓⇓⇓⇓⇓⇓⇓⇓⇓⇓⇓⇓-60]^，文献筛选流程图详见图1。

**图1:**
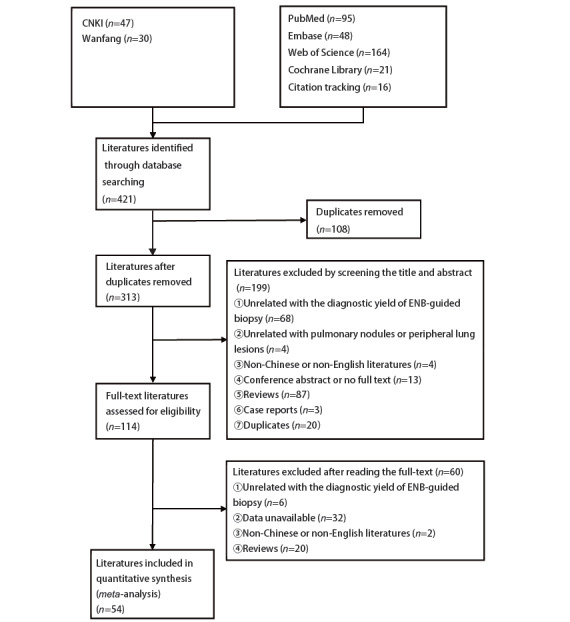
文献筛选流程图

### 1.4 资料提取

制定详细的文献信息摘录表，包括相关文献的基本信息（发布作者所在地区、发布时间、研究类型、研究对象、平均年龄、肺结节数量、平均病变大小、病变位置、病变类型）和纳入meta分析研究的主要特征（辅助技术、麻醉类型、采样技术、并发症类型）。对所有针对肺结节诊断的相关文献进行系统汇总。

### 1.5 统计分析

所有分析均使用Stata 16.0、RevMan 5.4和Meta-Disc 1.4软件进行，通过计算每项研究的灵敏度、特异度、阳性似然比（positive likelihood ratio, PLR）、阴性似然比（negative likelihood ratio, NLR）和诊断比值比（diagnostic odds ratio, DOR）来确定ENB的诊断准确性。使用随机效应模型将灵敏度、特异度、PLR、NLR和DOR进行合并，并通过Stata 16.0软件绘制森林图，汇总受试者工作特征曲线（summary receiver operating characteristic curve, sROC）。通过Meta-Disc 1.4和Stata 16.0软件进行meta回归与亚组分析探讨异质性来源。使用RevMan 5.4软件对纳入的诊断性研究进行质量评价。

## 2 结果

### 2.1 纳入研究的特征

共纳入54篇文献，合计55项研究进行meta分析，文章发表时间范围为2005年-2022年。所纳入研究的基本特征见表1，按照研究类型、研究对象、患者例数、平均/中位年龄、结节数、病变大小、位置和类型等进行整理。55项研究中合计5,187个活检结节被纳入，其中32项是回顾性研究，23项是前瞻性研究；患者平均年龄为66岁；结节平均大小为21.4 mm。对于研究对象的选择，多数研究选择PPLs在软式支气管镜下无法观察或影像学检查为肺结节但需活检进一步确认的患者，肺结节病变多集中在右肺上叶，病变类型多为外周型。

**表1:** 纳入分析研究的基本特征

Study	Country	Study type	Patient selection	Participants (M/F)	Mean/Median age	No. of lung nodules	Mean diameter	Localization of lesion	Type of lesion
Becker, 2005^[7]^	Germany	Pro	PPLs beyond the field of FB, regardless of lesion size	30 (23/7)	65 yr	30	39.81 mm	RUL (11); RML (2); RLL (3); LUL (9); LLL (5)	Peripheral PNs
Hautmann, 2005^[8]^	Germany	Pro	PPLs beyond the field of FB	16 (10/6)	63.7 yr	16	22 mm	RUL (5); RLL (4); LUL (4); LLL (3)	Peripheral PNs
Gildea, 2006^[9]^	USA	Pro	PPLs difficult to reach with standard bronchoscopic technique	58 (35/23)	67.9 yr	54	22.8 mm	RUL (18); RML (4); RLL (11); LUL (17); LLL (6)	Peripheral PNs
Schwarz, 2006^[10]^	Israel	Pro	PPLs beyond the field of FB, regardless of lesion size	15	NA	13	33.5 mm	RUL (3); RML (1); RLL (5); LUL (4)	Peripheral PNs
Eberhardt, 2007 (1)^[11]^	USA & Germany	Pro	PPLs beyond the field of FB	39 (20/19)	55 yr	39	28 mm	RUL (15); RML (3); RLL (6); LUL (7); LLL (8)	Peripheral PNs
Eberhardt, 2007 (2)^[11]^	USA & Germany	Pro	PPLs beyond the field of FB	40 (25/15)	51 yr	40	24 mm	RUL (11); RML (2); RLL (9); LUL (9); LLL (9)	Peripheral PNs
Eberhardt, 2007^[12]^	USA & Germany	Pro	PPLs beyond the field of FB	89 (50/39)	67 yr	92	24 mm	Upper lobes (52); Middle lobes (8); Lower lobes (32)	Peripheral PNs
Makris, 2007^[13]^	France	Pro	PPL located beyond the visible range of FB, suspicion of cancer by CT/PET scan, suggestive malignancy, after TTNA and MLN-TBNA, high-risk surgery	40 (30/10)	60 yr	40	23.5 mm	LUL+RUL (27); others (13)	Peripheral PNs
Wilson, 2007^[14]^	USA	Retro	PPL difficult to reach with standard bronchoscopic technique	248 (122/126)	63.1 yr	279	21 mm	RUL (85); RML (26); RLL (55); LUL (55); LLL (48); Lingula (8)	Peripheral PNs
Bertoletti, 2009^[15]^	France	Pro	PET-positive PPL beyond the field of FB, high-risk surgery	54 (47/7)	67 yr	54	31.2 mm	NA	Peripheral PNs
Lamprecht, 2009^[16]^	Austria	Retro	PPL beyond the field of FB and/or too small to be visible on fluoroscopy	13 (10/3)	64.2 yr	13	30 mm	RUL (5); RML (1); RLL (2); LUL (3); LLL (2)	Peripheral PNs
Eberhardt, 2010^[17]^	USA & Germany	Pro	Referral for small PPL suggestive of malignancy	54 (40/14)	65.1 yr	55	23.3 mm	left lung (56%); Upper lobes (60%)	Peripheral PNs
Seijo, 2010^[18]^	Spain	Pro	PPL, straightforward surgery or TTNA deemed suboptimal	51 (37/14)	62 yr	51	25 mm	Upper lobes (31); Middle lobe and lingula (6); Lower lobes (12)	NA
Mahajan, 2011^[19]^	USA	Retro	PPL beyond the field of FB, high-risk surgery	48	NA	48	20 mm	RUL (18); RML (3); RLL (8); LUL (10); LLL (8); Lingula (1)	Peripheral PNs
Brownback, 2012^[20]^	USA	Retro	PPL not accessible by conventional bronchoscopy	55 (33/22)	63 yr	55	30 mm	RUL (13); RML (5); RLL (10); LUL (21); LLL (4); Right hilar area (2)	Central PNs (15); intermediate PNs (26); peripheral PNs (14)
Study	Country	Study type	Patient selection	Participants (M/F)	Mean/Median age	No. of lung nodules	Mean diameter	Localization of lesion	Type of lesion
Pearlstein, 2012^[21]^	USA	Retro	PPL suggestive of malignancy based on CT and PET scan, unsuitable for TTNA, high-risk surgery, no other available biopsy site	104 (64/40)	69 yr	104	28 mm	NA	Peripheral PNs
Balbo, 2013^[22]^	Italy	Retro	Suspicion of cancer by CT/PET scan, suggestive malignant pulmonary nodule	40 (30/10)	71.5 yr	41	23.5 mm	Upper/middle lobes or lingula (24); Lower lobes (17)	Peripheral PNs
Mohanasundaram, 2013^[23]^	USA	Retro	Patients with suspected PPLs	41 (40/1)	65 yr	47	30.1 mm	RUL (17); RML (2) RLL (5); LUL (8); LLL (14); Lingula (1)	Peripheral PNs
Chen, 2014^[24]^	China	Pro	Suggestive of SPN on Helical CT	17 (11/6)	53 yr	20	18 mm	NA	Peripheral PNs
Loo, 2014^[25]^	USA	Retro	Patients with suspected PPLs	40 (12/28)	67 yr	50	26 mm	NA	Peripheral PNs
Odronic, 2014^[26]^	USA	Retro	PPL difficult to reach with standard bronchoscopic technique	91 (44/47)	66 yr	95	27 mm	RUL (32); RML (8); RLL (14); LUL (28); LLL (11); Lingula (2)	Peripheral PNs
Bowling, 2015^[27]^	USA	Retro	Patients with suspected PPL based on clinical history and radiographic abnormalities	107	67 yr	120	NA	RUL (31); RML (4); RLL (32); LUL (30); LLL (23)	Peripheral PNs
Flenaugh, 2016^[28]^	USA	Retro	Patients with confirmed PPLs	44 (23/21)	62.4 yr	71	22.1 mm	RUL (19); RML (6); RLL (15); LUL (16); LLL (15)	Peripheral PNs
Garwood, 2016^[29]^	USA	Retro	Patients with confirmed PPL	90 (31/59)	65.6 yr	92	22.7 mm	RUL (32); RML (6); RLL (22); LUL (22); LLL (10);	Peripheral PNs
Ozgul, 2016^[30]^	Turkey	Pro	PPL or SPN beyond the field of FB	56 (50/6)	60 yr	56	30 mm	RUL (21); RML (2); RLL (9); LUL (16); LLL (8)	Peripheral PNs
Raval, 2016^[31]^	USA	Retro	Patients with suspected lung cancer	48 (27/21)	67.7 yr	61	19.3 mm	RUL (23); RML (3); RLL (16); LUL (11); LLL (8)	Peripheral PNs
Bowling, 2017^[32]^	USA	Retro	Patients with suspected lung cancer	14 (9/5)	58.6 yr	14	NA	RUL (3); RLL (5); LUL (5); LLL (1)	Peripheral PNs
Gu, 2017^[33]^	China	Pro	Patients with confirmed PPLs	78 (49/29)	53.52 yr	84	19 mm	RUL (35); LUL (22)	Peripheral PNs
Huang, 2017^[34]^	China	Pro	PPL beyond the field of FB	18 (12/6)	68 yr	30	NA	RUL (12); RLL (5); LUL (5); LLL (8)	Peripheral PNs
Mukherjee, 2017^[35]^	USA	Retro	Patients with confirmed PPLs	31	66 yr	31	18 mm	RUL (11); RML (2); RLL (8); LUL (6); LLL (2); Lingula (1); Left stump(1)	Peripheral PNs
Sun, 2017^[36]^	China	Pro	PPL beyond the field of FB	40 (17/23)	59 yr	40	21.1 mm	Upper lobes (20); Middle lobes/lingula (6); Lower lobes (14)	Central PNs (5); intermediate PNs (9); peripheral PNs (26)
Study	Country	Study type	Patient selection	Participants, (M/F)	Mean/Median age	No. of lung nodules	Mean diameter	Localization of lesion	Type of lesion
Cho, 2018^[37]^	South Korea	Retro	Patients who underwent an ENB procedure for a biopsy and/or localization of a pulmonary resection	30 (20/10)	64 yr	32	9 mm	RUL (8); RML (3); RLL (11); LUL (5); LLL (5)	Peripheral PNs
Panchabhai, 2018^[38]^	USA	Retro	Patients with confirmed PPL	10 (7/3)	64.3 yr	10	20.5 mm	NA	Peripheral PNs
Patrucco, 2018^[39]^	Italy	Retro	Consecutive patients with pulmonary nodules and masses undergoing ENB	113 (78/35)	72.4 yr	113	24.6 mm	Upper and middle lobes (91); Lower lobes (22)	Peripheral PNs
Pritchett, 2018^[40]^	USA	Retro	Patients with suspected PPLs	75 (39/36)	70 yr	93	16 mm	RUL (34) RML (7); RLL (6); LUL (32); LLL (14)	Peripheral PNs
Sato, 2018^[41]^	Japan	Retro	Patients with confirmed PPLs	35	NA	35	15.28 mm	RUL (9); RML (2); RLL (10); LUL (7); LLL (7)	Peripheral PNs
Sobieszczyk, 2018^[42]^	USA	Retro	Patients with suspected lung cancer	22 (8/14)	69 yr	22	21 mm	RUL (4); RML (2); RLL (2); LUL (11); LLL (3)	Peripheral PNs
Taton, 2018^[43]^	Belgium	Pro	Patients with confirmed PPLs	32 (18/14)	68 yr	32	16 mm	RUL (8); RML (1); RLL (8); LUL (7); LLL (8)	Peripheral PNs
Yu, 2018^[44]^	China	Pro	Patients with confirmed PPLs	43 (23/20)	54.52 yr	43	25.16 mm	NA	NA
Cheng, 2019^[45]^	China	Retro	Consecutive patients detected to have PNs by CT scan	99 (73/26)	69.1 yr	99	26 mm	Lower lobes (26)	Central PNs (50)
Folch, 2019^[46]^	USA	Pro	Patients with confirmed PPLs	1,215 (617/598)	67.6 yr	1,344	20 mm	RUL (420); RML (105); RLL (255); LUL (360); LLL (204)	Peripheral PNs
Liu, 2019^[47]^	China	Pro	Patients with PPL on CT scan	20 (11/9)	NA	20	NA	NA	NA
Aboudara, 2020^[48]^	USA	Retro	90 consecutives performed ENB cases	90 (38/52)	64.4 yr	101	15 mm	RUL (34); RML (12); RLL (19); LUL (19); LLL (13); Lingula (4)	Central PNs (28); Peripheral PNs (55)
Andersen, 2020^[49]^	Denmark	Pro	100 consecutives performed ENB cases	100 (41/59)	69 yr	109	21 mm	Upper lobes (70); Lower lobes (32); Middle lobes/Lingula (5); Hilus (2)	NA
Stenger, 2020^[50]^	Denmark	Retro	80 consecutives performed ENB cases	80 (41/39)	69 yr	81	15.5 mm	RUL (35); RML (5); RLL (10); LUL (20); LLL (11)	Peripheral PNs
Xue, 2020^[51]^	China	Retro	PPL beyond the field of FB, suggestive of SPN on CT scan	18 (10/8)	66.7 yr	21	17 mm	RUL (6); RML (3); RLL (4); LUL (8)	Peripheral PNs
Katsis, 2021^[52]^	USA	Retro	Patients underwent navigational bronchoscopy for biopsy of 1 or more peripheral lung lesions	324 (142/182)	64.24 yr	363	19 mm	RUL (111); RML (33); RLL (68); LUL (82); LLL (56); Lingula (7)	Peripheral PNs
Oh, 2021 (1)^[53]^	Korea	Retro	Patients with PNs on CT scan	90 (55/35)	66 yr	100	27.9 mm	RUL (28); RML (9); RLL (19); LUL (21); LLL (23)	Peripheral PNs
Oh, 2021 (2)^[54]^	Korea	Pro	Patients with suspected PPLs on CT scan	30 (21/9)	63 yr	30	25.2 mm	RUL (8); RML (4); RLL (5); LUL (9); LLL (4)	Peripheral PNs
Study	Country	Study type	Patient selection	Participants (M/F)	Mean/Median age	No. of lung nodules	Mean diameter	Localization of lesion	Type of lesion
Patrucco, 2021^[55]^	Italy	Retro	Consecutive patients with pulmonary nodules and masses undergoing ENB	77 (53/24)	72.59 yr	103	28.91 mm	RLL+LLL (21); RUL+LUL+Middle lobes (82)	Peripheral PNs
Wang, 2021^[56]^	China	Retro	Patients with PNs on CT scan	25 (14/11)	66.8 yr	37	23.3 mm	RUL (14); RML (0); RLL (8); LUL (8); LLL (6)	Peripheral PNs
Folch, 2022^[57]^	USA	Pro	1,388 consecutive performed ENB cases in 37 academic and community sites	1,388 (690/698)	NA	1,388	20 mm	Upper lobes (897)	Peripheral PNs
Kim, 2022^[58]^	Korea	Retro	Patients with lung lesion biopsy undergoing ENB	94 (63/31)	68.2 yr	94	34.3 mm	RUL (33); RML (5); RLL (15); LUL (18); LLL (23)	Peripheral PNs
Yu, 2022^[59]^	China	Retro	PPL difficult to reach with standard bronchoscopic technique	60 (23/37)	62.8 yr	76	18 mm	RUL (32); RML (6); RLL (12); LUL (14); LLL (12)	Peripheral PNs
Yutaka, 2022^[60]^	Japan	Retro	Preoperative diagnosis prior to curative resection, high-risk surgery	100 (52/48)	67.7 yr	100	19.4 mm	Right lung (59); left lung (41); Upper lobes (47); Middle lobes (9); Lower lobes (44)	NA

Pro: prospective; Retro: retrospective; PPLs: peripheral pulmonary lesions; CT: computed tomography; TTNA: transthoracic needle aspiration; MLN-TBNA: mediastinal lymph node transbronchial needle aspiration; PET: positron emission tomography; FB: flexible bronchoscopy; RUL: right upper lobe; RML: right middle lobe; RLL: right lower lobe; LUL: left upper lobe; LLL: left lower lobe; SPN: solitary pulmonary nodule; PNs: pulmonary nodules; NA: not available; M: male; F: female.

纳入分析的研究均使用ENB系统进行诊断，其他辅助技术的使用情况见表2。其中14项研究单独使用ENB进行诊断，41项研究辅以其他技术进行诊断，如透视检查、rEBUS及ROSE。目前纳入的研究中提及ENB操作的麻醉方式主要为清醒镇静麻醉（conscious sedation, CS）、全身麻醉（general anesthesia, GA）和局部麻醉（local anesthetic, LA）。

**表2:** 纳入meta分析研究的主要特征（方法与采样措施）

Study	Additional technique	Type of sedation	Sampling technique	Adverse
Becker, 2005^[7]^	rEBUS, fluoroscopy	GA	Forceps, brush, curette	PTX (1), self-limiting bleeding (3)
Hautmann, 2005^[8]^	Fluoroscopy	LA/IVS	Forceps, needle	NA
Gildea, 2006^[9]^	Fluoroscopy	CS	Forceps, brush, BAL, TBNA, TBB	PTX (2) [CTI (2)]
Schwarz, 2006^[10]^	Fluoroscopy	CS	Forceps, brush	NA
Eberhardt, 2007 (1)^[11]^	NA	GA/moderate sedation	Forceps	PTX (2)
Eberhardt, 2007 (2)^[11]^	rEBUS	GA/moderate sedation	Forceps	PTX (3)
Eberhardt, 2007^[12]^	NA	GA/moderate sedation	Forceps, brush, needle, washing	PTX (2), intub (related to sedation) (1)
Makris, 2007^[13]^	NA	GA	Forceps	PTX (3) [CTI (1)]
Wilson, 2007^[14]^	Fluoroscopy, ROSE	LA/CS	Forceps, needle, TBLB	NA
Bertoletti, 2009^[15]^	NA	LA/N_2_O/O_2_	Forceps, brush	PTX (2) [CTI (1)]
Lamprecht, 2009^[16]^	ROSE	GA	Forceps, brush, needle	NA
Eberhardt, 2010^[17]^	EBUS	GA	Forceps, needle	PTX (1)
Seijo, 2010^[18]^	NA	CS	Forceps, needle	NA
Mahajan, 2011^[19]^	Fluoroscopy	CS	Brush, BAL, TBLB	PTX (5)
Brownback, 2012^[20]^	Fluoroscopy, ROSE	GA	Forceps, brush, needle, washing	Respiratory failure (2)
Pearlstein, 2012^[21]^	ROSE	GA	Forceps, brush, needle	PTX (6)
Balbo, 2013^[22]^	Fluoroscopy, ROSE	GA/CS	Forceps, brush, needle, BAL	NA
Mohanasundaram, 2013^[23]^	ROSE	IVS	Forceps, brush, needle	PTX (6) [intub (2), CTI (3)]
Chen, 2014^[24]^	NA	IVS	Forceps	NA
Loo, 2014^[25]^	ROSE	GA/deep sedation	Brush, needle, TBLB	NA
Odronic, 2014^[26]^	ROSE	NA	Brush, needle, BAL	NA
Bowling, 2015^[27]^	Fluoroscopy	GA/IVS	Forceps, brush, needle, BAL, TBNA	PTX (3), intub (1), CTI (1)
Flenaugh, 2016^[28]^	rEBUS	LA/CS	Forceps, brush, needle	NA
Garwood, 2016^[29]^	rEBUS, ROSE	GA	Forceps, brush, needle, BAL	PTX (6)[CTI (5)], bleeding (4)
Ozgul, 2016^[30]^	rEBUS	CS	Forceps, brush, BAL	PTX (1)
Raval, 2016^[31]^	EBUS	Moderate sedation/CS	Forceps, TTNA	PTX (1)
Bowling, 2017^[32]^	Fluoroscopy	GA	Forceps, brush, needle	PTX (1)
Gu, 2017^[33]^	rEBUS	GA	Forceps, brush, needle	PTX (1), bleeding (1)
Huang, 2017^[34]^	rEBUS	GA	Forceps, brush, needle	PTX (1)
Mukherjee, 2017^[35]^	Fluoroscopy	GA	Brush, needle, BAL	PTX (2) [CTI (1)]
Sun, 2017^[36]^	Fluoroscopy, rEBUS	LA/moderate sedation	Brush, washing	NA
Cho, 2018^[37]^	ROSE	GA	Forceps, brush, needle	Bleeding (2)
Panchabhai, 2018^[38]^	Fluoroscopy, rEBUS, ROSE	GA	Forceps, brush, needle, BAL	NA
Patrucco, 2018^[39]^	Fluoroscopy, ROSE	GA/deep sedation	Forceps, needle, BAL, TBLB	NA
Pritchett, 2018^[40]^	Fluoroscopy, ROSE	GA	Forceps, brush, needle, BAL	PTX (3)
Sato, 2018^[41]^	Fluoroscopy, ROSE	CS	Brush	PTX (1) [CTI (1)], hemopneumothorax (1)
Sobieszczyk, 2018^[42]^	Fluoroscopy, rEBUS	GA	Forceps, brush, needle	NA
Taton, 2018^[43]^	Fluoroscopy, rEBUS	GA	TBLC, forceps, TBB	PTX (1), bleeding (15)
Yu, 2018^[44]^	NA	GA	NA	NA
Cheng, 2019^[45]^	rEBUS, fluoroscopy	IVS/LA	Brush, needle, BAL	PTX (1), bleeding (1), respiratory failure (1)
Folch, 2019^[46]^	rEBUS, linear EBUS, fluoroscopy, ROSE	GA/moderate sedation	Forceps, brush, needle	PTX (52)
Liu, 2019^[47]^	NA	LA/GA	Brush, needle, BAL, TBLB	NA
Study	Additional technique	Type of sedation	Sampling technique	Adverse
Aboudara, 2020^[48]^	ROSE	NA	BAL, TBNA	PTX (2) [CTI (1)], bleeding (1), respiratory failure (1)
Andersen, 2020^[49]^	NA	IVS	Forceps, brush, washing	PTX (3)
Stenger, 2020^[50]^	rEBUS, fluoroscopy	NA	Forceps, brush, needle	Intrapulmonary haemorrhage (1)
Xue, 2020^[51]^	NA	GA	Forceps	NA
Katsis, 2021^[52]^	rEBUS, fluoroscopy, ROSE	IVS	Forceps, brush, washing	PTX (8), respiratory failure (1)
Oh, 2021 (1)^[53]^	NA	Moderate sedation	Forceps, needle	PTX (3), bleeding (4)
Oh, 2021 (2)^[54]^	NA	IVS/CS	Forceps, needle	PTX (1) [CTI (1)]
Patrucco, 2021^[55]^	ROSE	GA	Forceps, BAL	PTX (1)
Wang, 2021^[56]^	rEBUS	GA	Forceps, brush, BAL	PTX (1)
Folch, 2022^[57]^	rEBUS, fluoroscopy, ROSE	GA	Forceps, brush, needle	PTX (65), bleeding (37), respiratory failure (8)
Kim, 2022^[58]^	NA	Moderate sedation	Brush, BAL, washing	PTX (4)
Yu, 2022^[59]^	rEBUS, ROSE	GA	Forceps, brush	PTX (8), bleeding (76)
Yutaka, 2022^[60]^	NA	CS	Forceps, brush, TBLB	PTX (2)

rEUBS: radial endobronchial ultrasound; ROSE: rapid on-site cytological evaluation; EUBS: endobronchial ultrasound; GA: general anesthesia; CS: conscious sedation; IVS: intravenous sedation; LA: local anesthesia; BAL: bronchoalveolar lavage; TBLB: transbronchial lung biopsy; TBB: transbronchial biopsy; TBNA: transbronchial needle aspiration; TBLC: transbronchial lung cryobiopsy; PTX: pneumothorax; CTI: chest tube insertion; intub: intubation.

对于ENB的安全性汇总见表2。55项研究中共发生205例气胸，总发生率为3.27%，其中17例术后需要胸管置入，3例插管引流，1例血气胸需要开胸手术与楔形切除，其他未报告需要特殊治疗。此外，145例报告了轻微或中度出血，13例报告了术后呼吸衰竭，1例报告了与镇静剂有关的插管引流。

### 2.2 诊断准确性

ENB在PPLs诊断中的灵敏度范围为0.43-1.00，特异度范围为0.35-1.00。其中合并灵敏度、特异度分别为0.77（95%CI: 0.73-0.81）和0.97（95%CI: 0.93-0.99）（图2），PLRs、NLRs、DORs分别为24.27（95%CI: 10.21-57.67）、0.23（95%CI: 0.19-0.28）和104.19（95%CI: 41.85-259.37），sROC曲线下面积（area under the curve, AUC）为0.90（95%CI: 0.87-0.92）（图3）。

**图2:**
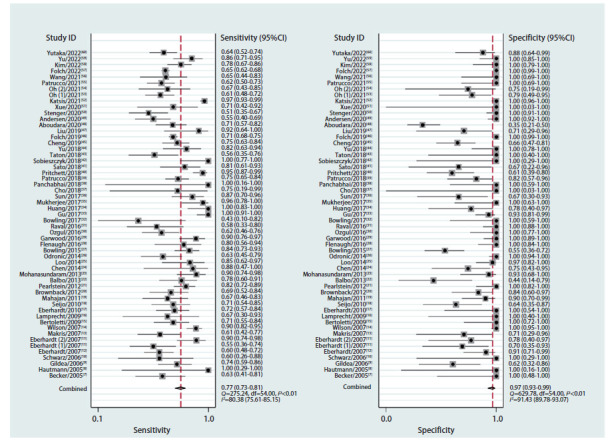
森林图（灵敏度与特异度）

**图3:**
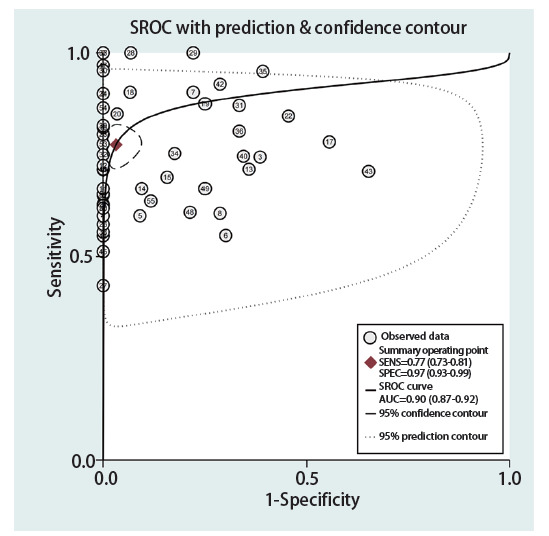
汇总受试者工作特征曲线（sROC）

### 2.3 质量评估

所有纳入研究均使用诊断试验准确性研究的偏倚评估工具QUADAS-2量表进行质量评价评估偏倚风险。按照评价条目，分别从病例的选择、待评价试验、金标准、病例流程和进展情况四个领域进行评估，并对前三个领域进行临床适用性评估。在54篇文献中，20篇在病例选择领域中报告了低风险偏倚。大多数文献在病例选择、待评价试验、金标准和病例流程与进展情况领域存在不明确的偏倚风险，因为没有使用合适的参考标准，而且不同研究对“导航成功”的定义不同，可能导致选择性报道。在临床适用性评价中，多数指南在病例选择和金标准领域中有较高的临床适用性。质量评价风险总结见图4。

**图4:**
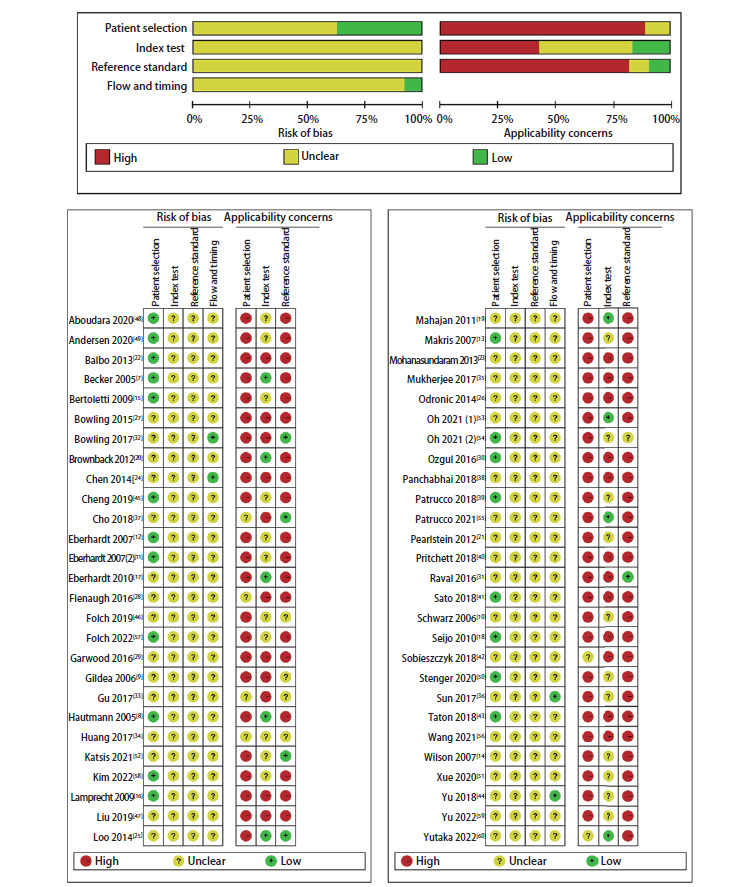
纳入研究的质量评价

### 2.4 亚组分析、meta回归与异质性检验

阈值效应是诊断试验中异质性的主要促成因素，利用Meta-Disc 1.4软件进行阈值效应检验，本研究灵敏度对数与（1-特异度）对数的Spearman相关系数为0.112（P=0.42），表明不能认为存在阈值效应。我们的分析显示ENB研究的合并灵敏度和特异度的Q检验P<0.01，且I^2^分别为80.38%和91.43%（图2），说明纳入的研究间存在显著的非阈值效应引起的异质性，使用随机效应模型合并。

为了确定纳入ENB研究的异质性来源，我们在此基础上利用Stata 16.0软件进行meta回归分析（表3），进一步从研究类型、辅助技术使用、样本量、病变大小和麻醉类型探寻异质性来源。结果显示，研究类型（P<0.01）、样本量（P=0.02）和麻醉类型（P<0.01）是诊断灵敏度异质性的主要来源；而对于特异度诊断，病变大小是异质性的主要来源（P=0.02）。当效应量为诊断比值比时，利用Meta-Disc 1.4软件对变量进行meta回归分析，结果表明，ENB诊断准确度与辅助技术使用和麻醉类型相关，使用辅助定位技术的诊断准确性是单独使用ENB的2.89倍[相对诊断比值比（relative diagnostic odds ratio, RDOR）=2.89（95%CI: 1.17-7.13），P=0.02]；CS的诊断准确性是GA的0.31倍[RDOR=0.31 (95%CI: 0.10-0.95), P=0.04]（表4）。

**表3:** Meta回归与亚组分析结果总结

Subgroups	Category	No. of studies	Sensitivity (95%CI)	P	Specificity (95%CI)	P
Design	Prospective	23	0.75 (0.68-0.81)	<0.01	0.96 (0.87-0.99)	0.39
	Retrospective	32	0.79 (0.74-0.84)		0.98 (0.92-0.99)	
Additional technique	Yes	41	0.80 (0.75-0.85)	0.07	0.98 (0.94-1.00)	0.23
	Individual ENB	14	0.67 (0.61-0.79)		0.90 (0.79-0.95)	
Sample size	≥25	45	0.77 (0.72-0.81)	0.02	0.97 (0.92-0.99)	0.75
	<25	10	0.80 (0.65-0.90)		0.94 (0.65-0.99)	
Lesion size	≥18 mm	45	0.77 (0.72-0.81)	0.09	0.98 (0.94-0.99)	0.02
	<18 mm	6	0.74 (0.57-0.86)		0.89 (0.39-0.99)	
Type of sedation	Conscious sedation	8	0.67 (0.60-0.74)	<0.01	0.89 (0.71-0.97)	0.18
	General anesthesia	28	0.79 (0.72-0.85)		0.98 (0.93-1.00)	

**表4:** ENB诊断准确性的meta回归分析结果

Variable	Coefficient	Std Err	P	RDOR (95%CI)
Design	-0.16	0.44	0.71	0.85 (0.35-2.04)
Additional technique	1.06	0.45	0.02	2.89 (1.17-7.13)
Sample size	-0.01	0.67	0.99	0.99 (0.26-3.82)
Lesion size	0.44	0.68	0.53	1.55 (0.39-6.08)
Type of sedation	-1.16	0.55	0.04	0.31 (0.10-0.95)

RDOR: relative diagnostic odds ratio.

为进一步确认异质性来源，我们通过Stata 16.0软件按照以下亚组进行分析：（1）前瞻性与回顾性研究；（2）是否使用辅助技术；（3）样本大小（样本量<25例被认为是小样本）；（4）病变大小（≥18 mm vs <18 mm）；（5）麻醉类型（CS vs GA）。结果显示，研究设计的类型在亚组之间的合并灵敏度有显著差异，23项研究为前瞻性研究，该亚组合并灵敏度为0.75（95%CI: 0.68-0.81），I^2^=68.21%，32项回顾性研究中的诊断灵敏度0.79为（95%CI: 0.74-0.84），显著高于前瞻性研究（P<0.01）；使用辅助定位技术的研究中ENB诊断灵敏度与特异度并未显著高于单独使用ENB的研究（P>0.05）；45项研究为大样本研究，该亚组合并灵敏度为0.77（95%CI: 0.72-0.81），I^2^=82.71%，10项小样本研究中的诊断灵敏度为0.80（0.65-0.90），显著高于大样本研究（P=0.02）；病变大小对ENB诊断特异度的影响具有统计学意义（P=0.02），而且对于较大结节（≥18 mm），ENB的合并灵敏度和特异度均高于小结节；对于麻醉方式的应用，8项研究采用CS，28项研究采用GA，在GA下ENB的灵敏度显著高于CS（P<0.01）。

### 2.5 发表偏倚

应用Deeks法漏斗图进行发表偏倚检验（图5），结果显示，纳入的研究间可能存在发表偏倚（P<0.01）。

**图5:**
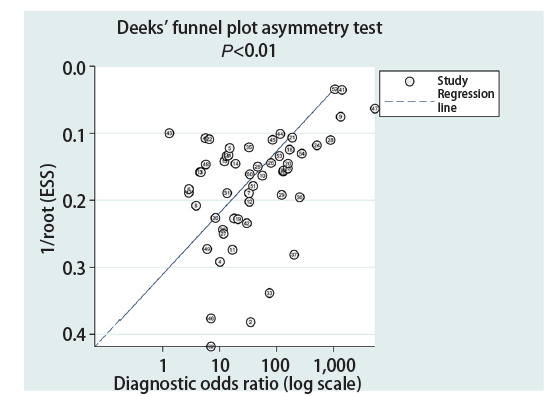
Deeks漏斗图评估发表偏倚

## 3 讨论

在本项针对ENB在PPLs诊断中准确性和安全性的meta研究中，共纳入55项研究，结果表明，ENB对PPLs的诊断具有良好的灵敏度（0.77）和特异度（0.97），具有较高的诊断价值；ENB引导下诊断试验的判别能力较好（合并DOR为104.19）；此外，ENB对PPLs的诊断具有较高的诊断准确性（AUC=0.90）。

ENB成功导航与诊断率之间的差异可能与ENB系统操作原理相关，ENB数据是基于虚拟空间重建的，因此在某些情况下可能会产生误导。可定位的实际位置可能与虚拟位置有很大的不同，虽然成功导航，但电磁导航的虚拟位置和实际位置之间的不匹配会降低ENB的诊断率。除此之外，ENB诊断准确率可能还受到一些潜在因素影响，比如是否使用辅助技术、病变大小和麻醉类型等。

目前常用辅助技术包括rEBUS、ROSE及透视，我们的研究结果显示，与单独ENB相比，辅助技术的使用可以显著提高诊断准确度。rEBUS可以提供PPLs 360度的高分辨率视图，允许实时超声引导下行经支气管针吸活检，并可通过软式支气管镜插入，图像的分辨率<1 mm^[61]^，在取样前使用ENB，然后使用rEBUS来确认已经到达病变，使操作者在获取组织样本前可以更准确地识别PPLs^[62]^。在2007年Eberhardt^[11]^的研究中，两种技术联合的诊断率为88%，明显高于单独使用ENB，证明了ENB和rEBUS的联合可以提高诊断率，而且不增加并发症的发生风险；ROSE技术运用于操作现场，对部分取材进行初步诊断，实时反馈取样是否成功，进一步指导后续操作^[63]^。ROSE技术运用于活检中，可缩短操作时间、提高诊断率、避免创伤并降低并发症的发生风险^[64,65]^；透视检查主要是为了确认在交替使用活检工具和存在导管路径曲折时，扩展工作通道没有发生位移。2020年，Aboudara^[48]^的一项回顾性研究表明，与单独使用ENB相比，ENB与透视检查联合的诊断率提高了25%。

肺结节的大小是显著影响诊断结果的重要因素，我们的meta回归与亚组分析结果显示，较大的病灶（≥18 mm）比小病灶（<18 mm）具有更高的特异度（0.98 vs 0.89）。Ali等^[62]^的meta分析结果显示，对结节大小>20 mm的病灶诊断率明显更高。另一项meta分析^[66]^存在具有相似结论，针对≤20 mm和>20 mm大小的病变，诊断率分别为60.9%（95%CI: 54.0%-67.7%）和82.5%（95%CI: 78.6%-86.4%），解释了异质性的主要来源。

在使用ENB的情况下，目前还没有研究针对GA与CS进行直接比较，我们的亚组分析与meta回归结果显示，GA下进行ENB比CS下具有更高的灵敏度（0.79 vs 0.67）以及诊断比值比。Pearlstein等^[21]^在其研究中指出，在GA下进行手术可以减少患者的活动，从而减少患者的解剖结构与胸部CT之间的差异，为医生的ENB操作减少了干扰，进而提高诊断率。Gex等^[6]^在其研究中也曾指出GA使ENB具有更高的诊断率（GA 69.2% vs CS 57.5%）。

目前，很少有研究分析病灶密度与诊断率之间的关系，因此本研究尚未获取到相关结节性质的文献信息。在Wang等^[56]^的研究中，ENB结合rEBUS的组合方式在实性肺结节中实现了90.9%的诊断率，显著高于亚实性肺结节（50%）（P=0.014）。在实性肺结节中，rEBUS可以灵敏地检测到气道周围的异常回声，使ENB导航更加实时和准确；大多数亚实性肺结节（包含磨玻璃结节和部分实性肺结节）是起源于肺泡上皮细胞的早期肺腺癌，多数生长在气管周围的肺实质中，肿瘤细胞的比例很小，导致活检工具不能准确地到达病灶，这也可能是导致亚实性肺结节诊断率较低的原因。

除此之外，研究类型也是本研究异质性的潜在来源，回顾性研究的灵敏度显著高于前瞻性研究，回顾性研究虽然可纳入的信息较为全面，但仍有局限性，其限制了检测轻微并发症的能力，如亚临床气胸或出血，可能没有被记录下来而产生偏倚^[52]^。

本研究局限性：（1）不同研究对导航检测时间的报道不一致，因此未对其进行亚组分析与meta回归分析；（2）由于纳入亚组分析与meta回归分析的不是患者的具体数据，因此得到的结论为探索性结果，具有统计合理性，应进一步提供患者层面的数据，以评估这些变量潜在的预测价值；（3）部分研究对“导航成功”的定义不同，选择的金标准不同，可能导致选择偏倚；（4）本文纳入的现有研究整体质量偏低，可能是由于样本代表性差，导致选择性偏倚，以期今后更好的研究提供确凿证据支持；（5）本研究严格遵守QUADAS-2量表评价要求，对纳入研究进行质量评价，但由于质量评价中存在大量的主观问题，部分问题的评估可能无法做到十分准确；（6）本研究存在发表偏倚，纳入研究可能存在漏检、部分纳入的研究样本量较小、阴性结果不易发表等情况，可能造成了本研究出现发表偏倚；本研究在筛选文献初期删除了会议论文，也是导致发表偏倚出现的潜在原因。

本研究较以往研究纳入研究的数量以及纳入的变量更加丰富，增加了辅助技术使用与麻醉方式的meta回归以及亚组分析。同时，本研究将GA与CS对ENB诊断率的影响进行直接比较，找出了部分异质性来源，解释了影响ENB诊断准确性的潜在原因。

本研究通过meta分析发现ENB是一种准确且安全的PPLs的诊断方式，具有较高的灵敏度和特异度，而且辅助定位技术和全身麻醉方式的使用进一步提高了ENB在PPLs中的诊断效果。此外，ENB术中不良事件和并发症的发生率低。我们通过汇总已发表的ENB诊断价值和安全性的证据，梳理了ENB在早期肺癌识别和诊断中的表现，期待今后大规模多中心的随机对照试验或者稳健的前瞻性研究为ENB的临床价值提供更加可靠的证据支持。
